# In Vitro Biological Activity and Lymphoma Cell Growth Inhibition by Selected Mexican Medicinal Plants

**DOI:** 10.3390/life13040958

**Published:** 2023-04-06

**Authors:** Nancy E. Rodríguez-Garza, Ramiro Quintanilla-Licea, César I. Romo-Sáenz, Joel H. Elizondo-Luevano, Patricia Tamez-Guerra, Cristina Rodríguez-Padilla, Ricardo Gomez-Flores

**Affiliations:** 1 Departamento de Microbiología e Inmunología, Facultad de Ciencias Biológicas, Universidad Autónoma de Nuevo León, San Nicolás de los Garza 66455, N.L., Mexico; 2 Grupo de Enfermedades Infecciosas y Tropicales (e-INTRO), IBSAL—CIETUS (Instituto de Investigación Biomédica de Salamanca—Centro de Investigación de Enfermedades Tropicales de la Universidad de Salamanca), Facultad de Farmacia, Universidad de Salamanca, 37007 Salamanca, Spain; 3 Departamento de Química, Facultad de Ciencias Biológicas, Universidad Autónoma de Nuevo León, San Nicolás de los Garza 66455, N.L., Mexico

**Keywords:** antitumor activity, cancer, ethnobotany, lymphoma, medicinal plants, natural extracts, Mexican plants

## Abstract

Cancer is a major health problem with significant morbidity and mortality. In addition, plants are a source of metabolites with diverse biological properties, including antitumor potential. In this study, we investigated the in vitro murine lymphoma L5178Y-R cell growth inhibition, human peripheral blood mononuclear cells (PBMC) toxicity and proliferation, and antioxidant, hemolytic, and anti-hemolytic activities of methanol extracts from 15 plants of traditional use in Mexico. *Justicia spicigera* caused the highest tumor cell growth inhibition with a half maximal inhibitory concentration (IC_50_) of 29.10 µg/mL and a selectivity index >34.36 compared with those of PBMC, whereas *Mimosa tenuiflora* showed the highest lymphoproliferative activity from 200 µg/mL compared with that induced by concanavalin A. In addition, *M. tenuiflora* showed an antioxidant effect (IC_50_ = 2.86 µg/mL) higher than that of ascorbic acid. Regarding the hemolytic and anti-hemolytic activity, all extracts presented significant anti-hemolytic activity. The extract of *J. spicigera* is emerging as a possible source of effective antineoplastic compounds.

## 1. Introduction

Cancer is a group of diseases that are characterized by uncontrolled and abnormal cell growth, as well as the potential to invade healthy tissues through metastasis [1]. This is a critical public health problem, causing significant morbidity and mortality [2]. In 2020, there were 19.3 million new cancer cases and 10 million deaths [3]. To date, more than 100 distinct types of cancer are known, which are classified according to the type of cells that were initially affected [1].

Lymphomas derive from T and B lymphocytes or natural killer cells, usually resulting in lymph node enlargement. Therefore, developing new, safe, and more specific biological targets is essential, especially for the most aggressive tumors [3]. In most types of cancers, chemotherapy is the treatment of choice. However, the presence of cancer cells resistant to chemotherapeutic agents [4], as well as the serious side effects they generate in patients, makes it essential to search for new drugs that are more effective and less toxic to patients [5].

Medicinal plants are an important source of metabolites with diverse biological properties that are used as active principles for the treatment of diseases [6]. They have been used for centuries and the World Health Organization recognizes their relevance in public health [7]. In recent years, plant secondary metabolites such as flavonoids, alkaloids, terpenoids, and saponins, among others, have been shown to be potent anticancer agents [8]. Some of the commonly used antineoplastics that have been identified and purified from plants [9] include Paclitaxel (Taxol^®^), which is a diterpene found in bark extracts from *Taxus brevifolia,* and vincristine, which is an alkaloid extracted from *Vinca rosea*. Another example is strigolactones, which are a group of phytohormones from *Striga* spp. and *Orobanche* spp. parasitic plants [10]. Mexico has a significant plant biodiversity, and more than 15 million people use traditional medicine [11]. In this country, more than 4500 plants have been traditionally used to treat various diseases, including cancer [12].

The aim of the present study was to evaluate the in vitro antitumor potential of methanol extracts from selected Mexican medicinal plants against the murine lymphoma cell line L5178Y-R, as compared with normal human peripheral blood mononuclear cells (PBMC), to determine their selectivity indices (SI). We used 15 plants of traditional medicinal use in Mexico, which belong to the families Acanthaceae, Anacardiaceae, Celastraceae, Compositae, Euphorbiaceae, Leguminosae, Papaveraceae, Poaceae, Rutaceae, Smilacaceae, and Zygophyllaceae, whose selection was based on the amount of reports of plants with anticancer activity [13]. In addition, PBMC proliferation, and antioxidant, hemolytic, and anti-hemolytic activities were evaluated.

## 2. Materials and Methods

### 2.1. Plant Material

Plants were purchased from Pacalli^®^ (pacalli.com.mx; Monterrey, Mexico). One specimen of each plant was identified by Professor Dr. Marco Antonio Guzmán-Lucio, curator of the Herbarium of Facultad de Ciencias Biológicas (FCB) at Universidad Autónoma de Nuevo León (UANL), México, where they were assigned voucher numbers (Table 1). Botanical names and families of plant species have been taxonomically validated, using ThePlantList website (http://www.theplantlist.org (accessed 21 January 2023)).

### 2.2. Plant Extracts Preparation

Plant extraction was performed by placing 25 g of each plant (dried and ground) in a Soxhlet extractor and 500 mL of absolute methanol (CTR Scientific, Monterrey, N.L., México) as extraction solvent. Extraction was maintained for 48 h, after which extracts were filtered and concentrated by vacuum evaporation with a rotary evaporator (Buchi R-3000; Brinkman Instruments, Inc., Westbury, NY, USA). Solvent was removed with a SpeedVac SPD121P concentrator (Thermo Fisher Scientific, San Jose, CA, USA) at 35 °C [14,15]. The extraction yield for each of the extracts was calculated by the following Formula (1):

(1) $$\%\;Yield=\frac{Final\;weight\;of\;dry\;extract}{Initial\;weight\;of\;the\;plant}\times 100$$

Next, 100 mg of each extract was dissolved in one milliliter of dimethyl sulfoxide (DMSO; Sigma-Aldrich, St. Louis, MO, USA), sterilized by filtration, using 0.22 µm pore size-membrane filters (Corning Incorporated, Corning, NY, USA), and stored at −20 °C until use. The final concentration of DMSO used in cell cultures was less than 1% (*v*/*v*), which did not affect cell viability [15].

#### Phytochemical Assay

Each of the crude plant extracts underwent phytochemical screening. The appearance of solids or foam during the reactions allows a semiquantitative evaluation of the presence of secondary metabolites [16]. We evaluated the presence of alkaloids, carbohydrates, coumarins, unsaturations (double bonds), flavonoids, quinones, saponins, sesquiterpene lactones, sterols, and tannins (phenolic groups). These tests were reported as presence (+) or absence (−) of compound groups. Protocols for each test are found as Supplementary Material (Supplementary Material S1: Phytochemical Screening). Solvents and chemicals used in phytochemical screening were purchased from Sigma-Aldrich.

### 2.3. Cell Lines and Cell Culture Conditions

Murine L5178Y-R lymphoma cells (ATCC CRL-1722) and PBMC were used in this study. PBMC were obtained from a 50 mL to 60 mL blood sample from a healthy donor, using Ficoll-Paque PLUS (GE Healthcare Life Sciences, Pittsburgh, PA, USA) and following supplier’s instructions. L5178Y-R cells and PBMC were maintained in RPMI 1640 culture medium (Life Technologies, Grand Island, NY, USA), supplemented with 10% heat-inactivated fetal bovine serum (FBS; Life Technologies) and 1% antibiotic/antifungal solution (Life Technologies) (referred as complete 1640 medium) at 37 °C in an atmosphere of 5% CO_2_ in air [17].

### 2.4. Effect of Extracts on Cell Growth

#### 2.4.1. Antitumor Activity of Plant Methanol Extracts

Cells were incubated in round-bottomed 96-well microplates (Corning Incorporated, Corning, NY, USA) at concentrations of 1 × 10^4^ L5178Y-R cells/well and 1 × 10^5^ PBMC/well in complete RPMI 1640 medium. After 24 h of incubation, cells were treated with methanol extracts at concentrations ranging from 3.9 µg/mL to 1000 µg/mL. The antineoplastic vincristine sulfate (VC) (Hospira, Warwickshire, UK) at 100 µg/mL was used as a positive control and untreated culture medium was used as a negative control [15]. Cells were incubated for 48 h at 37 °C in an atmosphere of 5% CO_2_ in air and cell viability was determined using the 3-[4,5-dimethylthiazol-2-yl]-2,5-diphenyltetrazoliumbromide (MTT; Affymetrix, Cleveland, OH, USA) colorimetric method by adding 15 µL of MTT/well (0.5 mg/mL final concentration) and incubating the plate at 37 °C for 3 h [18]. Plates were then decanted, formazan crystals were dissolved with 100 µL of DMSO, and optical densities (OD) were measured at 570 nm in a microplate reader (MULTISKAN GO; Thermo Fisher Scientific, Waltham, MA, USA). Percentage growth inhibition was calculated as follows (2):

(2) $$\%\;Cell\;growth\;inhibition=100-\left(\frac{{OD}_{570}\;Treated\;cells}{{OD}_{570}\;Untreated\;cells}\times 100\right)$$

#### 2.4.2. Determination of the Selectivity Index

Selectivity indices (SI) were determined to assess cytotoxic potential in tumor cells relative to toxicity in normal cells, where high SI indicates high potency and low cell toxicity [19]. In our study, it was considered that samples with SI values greater than three possess a high selectivity to tumor cells, according to Bezivin et al. (2003) [20]. Plant extract SIs were calculated by dividing the IC_50_ of normal cells (PBMC) by that of tumor cells (L5178Y-R), using the following Formula (3):

(3) $$SI=\frac{{IC}_{50}\;Normal\;cells}{{IC}_{50}\;Tumor\;cells}$$

#### 2.4.3. Effect of Plant Extracts on PBMC Lymphoproliferation

PBMC were incubated in round-bottomed 96-well microplates (Corning Incorporated) at 1 × 10^5^ cells/well in complete RPMI 1640 medium. After 24 h of incubation, cells were treated with methanol extracts at concentrations ranging from 100 µg/mL to 500 µg/mL, using 5 µg/mL concanavalin A (Con A) as a positive control and untreated culture medium as a negative control. Cells were incubated for 48 h at 37 °C in an atmosphere of 5% CO_2_ in air, and cell viability was determined using the colorimetric MTT reduction assay, as explained above [18]. Results were expressed as the proliferation index, which was calculated using the following Formula (4) [21]:

(4) $$Proliferation\;index=\frac{{OD}_{570}\;Treatment-{OD}_{570}\;Negative\;control}{{OD}_{570}\;Negative\;control}$$

In addition, we determined the half maximal effective concentration (EC_50_) of plant extracts for PBMC proliferation. The percentage of proliferation was calculated by multiplying the proliferation index by 100 [22].

#### 2.4.4. Synergistic Antitumor Activity of Plant Extracts

We evaluated the antitumor potential of combinations of bioactive extracts, as previously reported by Shang et al. (2019) [23]. We selected four plants that showed the highest percentage of tumor cell growth inhibition (IC_50_ < 50 µg/mL) against L5178Y-R murine lymphoma cells to determine if the combination of these extracts shows synergistic activity. For the assay, 1 × 10^4^ L5178Y-R lymphoma cells/well were incubated in round-bottomed 96-well plates in complete RPMI 1640 medium at 37 °C in 5% CO_2_ in air for 24 h, after which cells were treated with plant methanol extracts, alone or in combination, at concentrations ranging from 7.8 µg/mL to 125 µg/mL, using 100 µg/mL VC as a positive control and untreated culture medium as a negative control. After 48 h of incubation at 37 °C in 5% CO_2_ in air, cell viability was determined by the colorimetric MTT reduction assay, as explained above [18]. The type of interaction that occurred between crude methanol extracts was determined using the Synergy Finder 3.0 application (https://synergyfinder.fimm.fi/) (accessed 30 November 2022). Scores obtained in this application indicate the following characteristics: (a) the interaction is likely to be antagonistic, when the score is lower than −10, (b) the interaction is likely to be additive, when the score ranges from −10 to 10, and (c) the interaction is likely to be synergistic, when the score is higher than 10 [24].

### 2.5. Antioxidant Activity

To determine the antioxidant activity of plant extracts, we incubated 100 μL of the extracts at concentrations ranging from 3.9 µg/mL to 500 µg/mL plus 100 μL of a 0.1 mM 2,2-diphenyl-1-picrylhydrazil methanolic solution (DPPH; Sigma-Aldrich) in flat-bottomed 96-well microplates for 30 min at room temperature in darkness, after which ODs were determined at 517 nm. DMSO was used as a blank and ascorbic acid (J. T. Baker, Phillipsburg, NJ, USA) as a positive control at concentrations ranging from 0.2 µg/mL to 250 µg/mL [25]. Percentage inhibition of DPPH was calculated using the following Formula (5):

(5) $$\%\;inhibicion\;DPPH=\frac{{OD}_{517}\;Treatment-{OD}_{517}\;Negative\;control}{{OD}_{517}\;Negative\;control}\times 100$$

### 2.6. Hemolytic and Anti-Hemolytic Activity

We obtained 20 mL of blood from a healthy volunteer in tubes with anticoagulant (BD Vacutainer K2 EDTA; Becton Dickinson & Company, Franklin Lakes, NJ, USA). Red blood cells were washed three times with phosphate buffered saline solution (PBS, pH 7.2) and a 5% erythrocytes suspension was prepared in sterile PBS. For the evaluation of the hemolytic activity in 2 mL tubes, we incubated extracts at concentrations ranging from 200 µg/mL to 1000 µg/mL and 5% erythrocytes suspension for 30 min at 37 °C, after which they were centrifuged at 4 °C for 5 min at 13,000 rpm [26], using distilled water as a positive control for hemolysis and PBS as a negative control. For the evaluation of the antihemolytic effect, we incubated 2,2′-azobis(2-amidinopropane) dihydrochloride (AAPH), which was prepared in PBS at a concentration of 150 mM extract and a erythrocyte suspension for 5 h at 37 °C at 200 rpm in darkness using a rotating incubator (MaxQ, Thermo-Scientific, Waltham, MA, USA), and using PBS with the erythrocyte suspension without AAPH as the negative control and the erythrocyte suspension with 150 mM AAPH as a positive control (C+) [27]. In both cases, 200 µL of the supernatant was taken from each tube and transferred to a flay-bottomed 96-well microplate to measure the OD of the released hemoglobin at 540 nm in a microplate reader [28]. The percentage of hemolysis and anti-hemolytic activity for each sample was calculated using the following Formulas (6) and (7):

(6) $$\%\;Hemolysis=\frac{{OD}_{540}\;Treatment-{OD}_{540}\;Negative\;control}{{OD}_{540}\;Positive\;control-{OD}_{540}\;Negative\;control}\times 100$$

(7) $$\%\;AAPH\;Inhibition=1-\left(\frac{{OD}_{540}\;Treatment-OD\;Negative\;control}{{OD}_{540}\;Positive\;control-OD\;Negative\;control}\times 100\right)$$

### 2.7. Ethical Statement

The study with human PBMC and erythrocytes was performed under the approval of the Ethics Committee of FCB-UANL (Registration Number CI-08-2020) and under the consent of a healthy donor (the Letter of Informed Consent for Donors of Human Biological Sample Material and the Institutional Review Board Approval are attached as Supplementary Material), following the provisions of the Official Mexican Technical Standard NOM-253-SSA1-2012. We did not develop studies involving animals.

### 2.8. Statistical Analysis

Data represent the mean ± SD of triplicate determinations of at least three independent experiments. A one-way analysis of variance was used to determine the significant difference between the tested concentrations. Tukey’s post hoc test was used to determine the difference between the treatment means. The Probit test was used to calculate the IC_50_ (half maximal inhibitory concentration) and the EC_50_ (half maximal effective concentration) values. Statistical analyses were performed using the GraphPad Prism 8 statistical package (GraphPad Software Inc., San Diego, CA, USA).

## 3. Results and Discussion

### 3.1. Plant Material Identification

Table 1 provides specific information on medicinal plants used in the present study. Of the fifteen plants evaluated, five belong to the Compositae family, and the other plants belong to different families. Plant methanol extracts were analyzed to determine their cytotoxic, lymphoproliferative, antioxidant, hemolytic, and anti-hemolytic activities, and their synergistic antitumor potential of the most active extracts against L5178Y-R tumor cells. These plants have been demonstrated their antitumor properties [13].

### 3.2. Plant Extract Yields and Phytochemical Analysis

Table 2 shows yields (11.14% to 27.37%) of plant methanol extracts used in this study. All extracts were positive for unsaturation (double bonds) and coumarins. *A. mexicana* and *R. chalepensis* were positive for alkaloids, whereas *J. spicigera*, *S. mexicanum*, *S. aspera*, and *T. lucida* extracts were negative for the sterol test, and only *P. obtusifolium* was positive for saponins. Plant extracts consist of a complex mixture of various compounds such as alkaloids, esters, aldehydes, carbohydrates, terpenes, and polyphenols, among others [29]. In addition, crude extracts, semi-purified fractions, and pure compounds have been used in different approaches testing biological activities [30]. However, it is necessary to search for new sources and specific compounds against cancer [14,31]. In this context, Mexico is an attractive country for its great variety of endemic plants [32].

### 3.3. Cell Growth Inhibition by Plant Extracts

We evaluated the percentage cell growth inhibition of plant extracts against the tumor cell line L5178Y-R and PBMC, using the colorimetric MTT reduction assay, which allowed us to determine the SI, as explained above [19]. Table 3 shows the effect of plant extracts on cell growth inhibition, where the most active extracts against L5178Y-R cells were *J. spicigera* (IC_50_ = 29.10 µg/mL), *A. ludoviciana* (IC_50_ = 32.39 µg/mL), *J. dioica* (IC_50_ = 39.25 µg/mL), and *M. tenuiflora* (IC_50_ = 47.10 µg/mL). The other extracts showed activities with IC_50_ values higher than that of *A. mexicana* (IC_50_ = 70.73 µg/mL). When the extracts were evaluated on PBMC viability, *J. spicigera*, *A. adstringens*, *S. mexicanum*, *A. ludoviciana*, *H. inuloides*, *P. decompositum*, *M. tenuiflora*, and *S. aspera* did not significantly alter PBMC viability, since they presented IC_50_ > 1000 µg/mL, thus indicating low toxicity for normal cells [31]. VC was also not toxic for PBMC. Previous studies with VC showed its potent anticancer effects on solid tumors, such as hepatocellular carcinoma, breast cancer, and lung cancer [33], in addition to the L5178Y-R cell line [34]. It has been well documented that VC has no cytotoxic effects on PBMC nor on normal L929 and Vero cells, suggesting a selective action of the drug against leukemic cells [14,17,35].

In the present study, we found antitumor potential of traditionally used plants in Mexico without affecting human PBMC. Therefore, with the results of cytotoxicity to PBMC, we determined the SI, where a value of 3 or greater was taken as the cut-off point [20]. In the present study, *J. spicigera*, *A. adstringens*, *S. mexicanum*, *A. ludoviciana*, *H. inuloides*, *P. decompositum*, *P. obtusifolium*, *J. dioica*, *M. tenuiflora*, and *S. aspera* extracts showed their following SIs: >34.36, >5.07, >7.41, >30.87, >7.14, >6.01, 3.99, >21.23, and >4.00, respectively (Table 3). Extracts with the lowest SI were *P. obtusifolium*, *T. lucida*, *C. citratus*, *R. chalepensis*, and *L. tridentata*, since they showed SI lower than 3. The SI is commonly used to measure the efficacy of drugs, where the reduction of cell survival is evaluated [36]. Thus, we determined the cytotoxic potential of each extract in a tumor cell line (L5178Y-R) in relation to the toxicity in normal cells (PBMC), where a high SI (greater than 3) indicates high potency against L5178Y-R cells and low cell toxicity to PBMCs [19].

### 3.4. PBMC Proliferation

**Figure 1:**
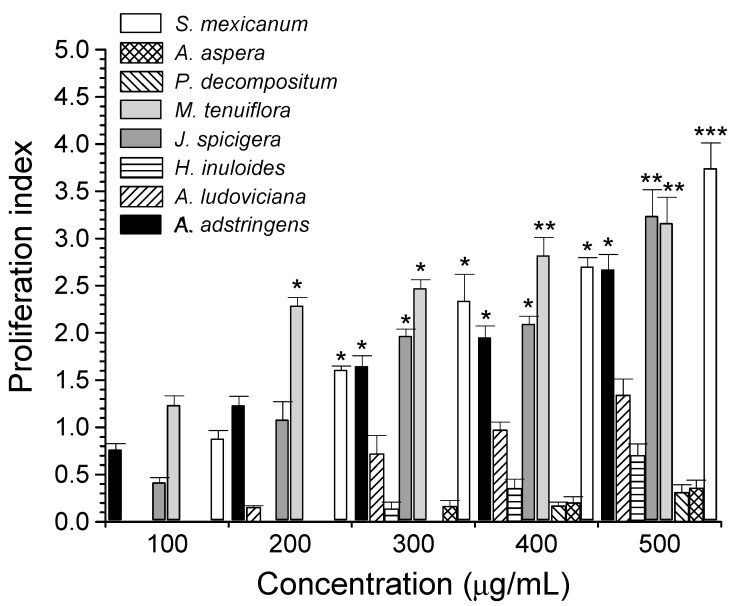
PBMC proliferation by Mexican plant extracts. Values represent the PBMC proliferation index ± SD by plant extracts, using 5 µg/mL Con A as a positive control. * *p* < 0.05, ** *p* < 0.01, *** *p* < 0.001, as compared with Con A (proliferation index = 1.21 ± 0.18).

Figure 1 shows the effect of plant extracts on PBMC proliferation. Extracts that showed the highest and significant cell proliferation activity were *M. tenuiflora* at concentrations ranging from 200 µg/mL to 500 µg/mL, followed by *S. mexicanum*, *A. adstringens*, and *J. spiciguera*. In addition, *A. ludoviciana*, *H. inuloides*, *P. decompositum*, and *S. aspera* extracts showed lower lymphoproliferative activity than that of Con A. The remaining extracts presented null activity at all concentrations tested, and they were not included in Figure 1. Crude plant extracts are complex mixtures that contain a great diversity of secondary metabolites that act synergistically to stimulate a biological response [37], for which the lymphoproliferative activity of PBMC is probably due to the synergistic effects of flavones, phenols, terpenes, sesquiterpenes, and tannins present in the extracts [38].

The proliferative activity of plant extracts on PBMC is shown in Table 4. *M. tenuiflora, A. adstringens*, and *S. mexicanum* extracts showed the highest proliferative effect on PBMC with EC_50_ of 14.93 µg/mL, 18.3 µg/mL, and 21.95 µg/mL, respectively.

The proliferative effect of *A. adstringens* may be due to the action of anacardic acids such as 6-pentadecyl salicylic acid (6-PSA), which are the main compounds of this plant. It has been shown that 6-PSA did not reduce PBMC cell viability, as compared with the gastric tumor cell line AGS, where it was determined that it showed cytotoxic and genotoxic activity and induced cell death by apoptosis in a caspase 8-dependent manner, thus evidencing its therapeutic potential [39]. In addition, it has been shown that an extract from *S. mexicanum* bark has antitumor activity against breast cancer cells (MDA-MB-231 and MCF7) but promotes proliferation of non-breast derived cells (MCF 10A) and PBMC, which may be due to the quinone triterpenes pristimerin and tingenone [40], suggesting that *S. mexicanum* cortex has a potential application in cancer treatment [41].

Different saponins isolated from *M. tenuiflora* possess a synergistic antitumor effect against the lymphoma cells RDM 4 and Molt 4, and have a significant mitogenic effect on mouse fibroblast cells LMTK and human fibroblasts [42,43]. This is the first report showing *M. tenuiflora* proliferation activity on PBMC.

### 3.5. Synergistic Antitumor Activity of Plant Extracts against L5178Y-R Lymphoma Cells

The combination of two or more therapeutic agents is a complementary approach in cancer therapy. Clinical studies have reported the beneficial effects of herbal medicines in the treatment and quality of life of cancer patients when used in combination with conventional therapy [44]. Plants selected for this study were *A. ludoviciana*, *J. dioica*, *J. spicigera*, and *M. tenuiflora* because they showed IC_50_ < 50 µg/mL against L5178Y-R cells. Table 5 shows the type of interaction presented by the various combinations of the most active extracts against L5178Y-R cells, where it can be seen that AlJd, AlJs, and JdJs combinations evidenced synergistic activity. In addition, AlMt and JsMt combinations showed additive activity. Only JdMt combination presented antagonistic activity. A series of scientific investigations have demonstrated the synergistic activity of plant extracts, for which the next step was to investigate a synergistic effect between the most active extracts. It is believed that the active compounds of plants modify and inhibit mechanisms of acquired resistance in cells, thus exhibiting a synergistic effect [45].

### 3.6. Antioxidant, Hemolytic, and Anti-Hemolytic Activities of Plant Extracts

Extensive research indicates that oxidizing agents such as reactive oxygen species (ROS) at low levels have beneficial effects on health. However, excessive accumulation causes various disorders, including carcinogenesis, as ROS play a crucial role in cancer cell survival [46]. In the present study, the antioxidant activity of plant extracts related to the DPPH free radical scavenging effect was compared with that of ascorbic acid, which showed an IC_50_ of 7.23 µg/mL. Extracts with an IC_50_ ≤ 50 µg/mL were considered to have relevant antioxidant activity [47]. *J. spicigera* (IC_50_ = 15.68 µg/mL), *A. adstringens* (IC_50_ = 17.22 µg/mL), *M. tenuiflora* (IC_50_ = 2.86 µg/mL), and S. *aspera* (IC_50_ = 29.21 µg/mL) extracts showed the highest antioxidant effect among all the extracts evaluated, with *M. tenuiflora* the extract that presented significantly (*p* < 0.001) higher antioxidant activity compared with the positive control, whereas *R. chalepensis* extract (IC_50_ = 859.85 µg/mL) showed the lowest antioxidant effect (Table 6). In general, a natural extract that may be used as a universal antioxidant does not exist. Therefore, it is necessary to screen for specific antioxidant compounds for certain outcomes [30]. Various authors have demonstrated the antioxidant activity of extracts from various plants through the DPPH test and have concluded that polyphenolic components and terpenes are the main source of antioxidant activity in various extracts, such as eucalyptus (*Eucalyptus camaldulensis* Dehnh.), which is probably due to gallic and ellagic acids [48]. Moreover, the resveratrol present in grapes (*Vitis vinifera* L.) has chemopreventive and therapeutic effects in reducing human breast, uterus, blood, prostate, and ovarian cancers, among others [49]. As mentioned above, *M. tenuiflora* extract maintained a high antioxidant activity, which was higher than that of the positive control. These results showed that one or several antioxidant compounds present in *M. tenuiflora* extract have the potential to act for the free radical scavenging activity (DPPH assay) through a hydrogen transfer reaction [50].

One of the research objectives of studying medicinal plants is to develop and use assays that are easy to use, reproducible, and inexpensive, such as the determination of the toxic activity of plant extracts, fractions, combinations, and/or formulations on human erythrocytes by the hemolysis test [51]. Therefore, we decided to evaluate the hemolytic potential of plant extracts and it was determined that none of the extracts was toxic to human erythrocytes, according to the criteria of López Villarreal et al. (2022) [29], and *A. adstringens* and *S. aspera* are the extracts with the lowest activity, with IC_50_ of 268.40 and 262.80 µg/mL, respectively. *S. mexicanum*, *A. ludoviciana*, *P. decompositum*, *P. obtusifolium*, *M. tenuiflora*, and *L. tridentata* extracts showed IC_50_ > 1000 (Table 6). The median lethal dose (IC_50_) for an extract to be considered non-toxic is ≥ 1000 µg/mL. Those between 500 µg/mL and 1000 µg/mL are considered slightly toxic, between 100 µg/mL and 500 µg/mL are considered moderately toxic, and between 10 µg/mL and 100 µg/mL are considered highly toxic [47]. In addition, the anti-hemolytic effect of the extracts was determined by the hemolysis test, using the AAPH radical to form peroxyl radicals and induce membrane oxidation in human erythrocytes [28]. We observed a significant anti-hemolytic effect for all extracts evaluated, as compared with the positive control. *A. adstringens*, *H. inuloides*, *P. decompositum*, and *S. aspera* extracts possessed the highest anti-hemolytic activity, with IC_50_ values of 1.07 µg/mL, 1.09 µg/mL, 1.18 µg/mL, and 0.88 µg/mL, respectively, which agrees with a report by Elizondo et al. (2022) [14].

Results on the hemolytic and anti-hemolytic potential of plant extracts emphasize the antioxidant properties of the extracts, since the study of the uptake of azo radicals, such as the AAPH radical, was designed to induce oxidative stress in the lipid and aqueous phases of cells and assess the vulnerability of erythrocytes to oxidative stress [52]. The breakdown of AAPH, which is soluble in water at physiological temperature, generates free radicals that attack the erythrocyte membrane and induce lipid peroxidation that leads to hemolysis [53].

Therefore, these results showed the safe use of medicinal plants and their doses [54]. Taken together, our study demonstrated the antitumor activity of extracts from plants of medicinal use in Mexico as well as their potential to induce PBMC proliferation plus antioxidant and anti-hemolytic activities on human erythrocytes.

## 4. Conclusions

*J. spicigera*, *A. ludoviciana*, *J. dioica*, and *M. tenuiflora* methanol extracts significantly inhibited L5178Y-R lymphoma cell growth. Moreover, *J. spicigera*, *A. ludoviciana*, and *M. tenuiflora* presented the highest SI when evaluated against PBMC, with SIs of >34.36, >30.87, and >21.23, respectively, making them promising candidates for further study against other cell lines and for bio-targeted purification of their most active components. It was also demonstrated that the extracts in combination showed in vitro synergistic activity when evaluated against L5178Y-R cells, which opens a new line of research regarding the possible combined use of the different plants. Overall, results of the present study validated the use of medicinal plant extracts and their combinations in cancer. In addition, the evaluation of extracts from plants used in traditional medicine reveals the potential of identifying bioactive compounds showing antitumor, antioxidant, and anti-hemolytic activity.

## 5. Future Perspectives

In a second stage of this project, we will develop a bio-directed fractionation of the active plant methanol extracts in the L5178Y-R lymphoma model, as well as the purification of the components with biological activity and determination of the mechanisms of molecular action. In addition, in vivo studies of the most promising extracts and active compounds are warranted to validate their potential use in L5178Y-R lymphoma treatment.

## Figures and Tables

**Table 1:** Taxonomic identification of medicinal plants used in this study.

Family	Scientific Name	Common Name	Used Part	Voucher Number
*Acanthaceae*	*Justicia spicigera* Schltdl.	Muicle	Stems, leaves, and flowers	30649
*Anacardiaceae*	*Amphipterygium adstringens* (Schltdl.) Standl.	Cuachalalate	Bark	30642
*Celastraceae*	*Semialarium mexicanum* (Miers) Mennega	Cancerina	Bark	30647
*Compositae*	*Artemisia ludoviciana* Nutt.	Estafiate	Stems, leaves and flowers	30643
*Compositae*	*Heterotheca inuloides* Cass.	Árnica	Flowers	30646
*Compositae*	*Psacalium decompositum* (A. Gray) H. Rob. and Brettell	Matarique	Roots	30652
*Compositae*	*Pseudognaphalium obtusifolium* (L.) Hilliard and B.L. Burtt.	Gordolobo	Flowers	30653
*Compositae*	*Tagetes lucida* Cav.	Hierbanís or Yerbaniz	Young leaves and stems	30656
*Euphorbiaceae*	*Jatropha dioica* Sessé	Sangre de Drago	Roots	30648
*Leguminosae*	*Mimosa tenuiflora* (Willd.) Poir.	Tepezcohuite	Bark	30651
*Papaveraceae*	*Argemone mexicana* L.	Chicalote	Leaves	29127
*Poaceae*	*Cymbopogon citratus* (DC.) Stapf.	Zacate limón	Young leaves and stems	30644
*Rutaceae*	*Ruta chalepensis* L.	Ruda	Young leaves and stems	30654
*Smilacaceae*	*Smilax aspera* L.	Zarzaparilla	Roots	30655
*Zygophyllaceae*	*Larrea tridentata* (Sessé and Moc. ex DC.) Coville	Gobernadora	Young leaves and stems	30650

**Table 2:** Plant extract yields and phytochemical screening.

Plant Extract	%	Chemical Groups									
		Alk	Carb	Cm	Db	Flv	Qn	Sp	Sl	St	Tn
*J. spicigera*	27.37	−	+	+	+	−	+	−	−	−	+
*A. adstringens*	24.82	−	+	+	+	+	+	−	+	+	+
*S. mexicanum*	11.14	−	+	+	+	−	+	−	+	−	−
*A. ludoviciana*	17.31	−	+	+	+	+	+	−	+	+	+
*H. inuloides*	21.21	−	+	+	+	+	−	−	−	+	+
*P. decompositum*	14.43	−	+	+	+	+	−	−	−	+	+
*P. obtusifolium*	16.99	−	+	+	+	+	+	+	+	+	+
*T. lucida*	20.63	−	+	+	+	+	−	−	+	−	+
*J. dioica*	19.58	−	+	+	+	−	+	−	−	+	−
*M. tenuiflora*	11.25	−	−	+	+	+	+	−	+	+	+
*A. mexicana*	11.26	+	−	+	+	+	−	−	+	+	−
*C. citratus*	23.04	−	+	+	+	+	−	−	−	+	−
*R. chalepensis*	19.40	+	+	+	+	+	−	−	+	+	+
*S. aspera*	18.26	−	+	+	+	−	+	−	+	−	−
*L. tridentata*	26.61	−	−	+	+	+	+	−	+	+	+

%: Extraction yield percentage; Alk: alkaloids, Carb: carbohydrates, Cm: coumarins, Db: Double bonds, Flv: flavonoids, Qn: quinones, Sp: saponins, Sl: sesquiterpene—lactones, St: sterols, Tn: tannins; (+): present, (−): absent.

**Table 3:** Effect of methanol plant extracts on L5178Y-R and PBMC viability.

Plant Extract	IC_50_ (µg/mL)		SI
	L5178Y-R	PBMC	
*J. spicigera*	29.10 ± 5.23 ^a^	>1000 ^†^	>34.36
*A. adstringens*	197.66 ± 4.07 ^e^	>1000 ^†^	>5.07
*S. mexicanum*	135.00 ± 5.19 ^c^	>1000 ^†^	>7.41
*A. ludoviciana*	32.39 ± 5.59 ^a^	>1000 ^†^	>30.87
*H. inuloides*	140.00 ± 10.03 ^cd^	>1000 ^†^	>7.14
*P. decompositum*	166.50 ± 19.19 ^d^	>1000 ^†^	>6.01
*P. obtusifolium*	710.55 ± 24.33 ^g^	745.40 ± 12.90 ^d^	1.05
*T. lucida*	251.17 ± 6.02 ^f^	732.05 ± 8.59 ^d^	2.91
*J. dioica*	39.25 ± 3.81 ^a^	156.60 ± 10.76 ^a^	3.99
*M. tenuiflora*	47.10 ± 10.19 ^ab^	>1000 ^†^	>21.23
*A. mexicana*	70.73 ± 2.36 ^b^	398.45 ± 7.97 ^c^	5.63
*C. citratus*	209.24 ± 6.15 ^e^	312.41 ± 2.87 ^b^	1.49
*R. chalepensis*	71.81 ± 1.86 ^b^	183.68 ± 11.89 ^ab^	2.56
*S. aspera*	250.00 ± 5.29 ^f^	>1000 ^†^	>4.00
*L. tridentata*	214.64 ± 1.63 ^e^	403.05 ± 13.72 ^c^	1.88

Data are mean ± SD of the IC_50_ (µg/mL) for each extract against the evaluated cell lines. Different letters within the same column are significantly different, analyzed by the post hoc Tukey test (*p* < 0.05). SI represents IC_50_ for the PBMC cell line divided by that of the L5178Y-R cell line after 72 h, as detailed in the Section 2. VS was used as a positive control at 0.05 µg/mL. ^†^ As IC_50_ was above 1000 µg/mL, these values were not considered for Tukey analysis.

**Table 4:** Lymphoproliferative activity of methanol extracts on PBMC cells.

Plant Extract	EC_50_ (µg/mL)
*J. spicigera*	154.14 ± 7.3 ^c^
*A. adstringens*	18.30 ± 1.5 ^a^
*S. mexicanum*	21.95 ± 5.6 ^ab^
*A. ludoviciana*	256.99 ± 11.78 ^d^
*H. inuloides*	431.69 ± 19.2 ^e^
*P. decompositum*	560.84 ± 42.01 ^f^
*P. obtusifolium*	ND
*T. lucida*	ND
*J. dioica*	ND
*M. tenuiflora*	14.93 ± 4.3 ^a^
*A. mexicana*	ND
*C. citratus*	ND
*R. chalepensis*	ND
*S. aspera*	595.76 ± 27.9 ^g^
*L. tridentata*	ND

Data are means ± SD of the EC_50_ (µg/mL) for each extract evaluated on PBMC cells. Different letters within the same column are significantly different, analyzed by the post hoc Tukey test (*p* < 0.05). ND: Not determined, because these extracts did not show lymphoproliferative activity at any of the evaluated concentrations.

**Table 5:** In vitro interaction between bioactive plant extracts on murine lymphoma cell growth.

Extracts Combination	Combination Code	HSA Model Value	Interaction
*A. ludoviciana* + *J. dioica*	AlJd	13.737	Synergism
*A. ludoviciana* + *J. spicigera*	AlJs	10.951	Synergism
*A. ludoviciana* + *M. tenuiflora*	AlMt	−8.663	Additive
*J. dioica* + *J. spicigera*	JdJs	18.111	Synergism
*J. dioica* + *M. tenuiflora*	JdMt	−19.151	Antagonism
*J. spicigera* + *M. tenuiflora*	JsMt	2.985	Additive

The value shown corresponds to that obtained with the HSA model, when evaluating concentrations from 7.8 µg/mL to 125 µg/mL of the extracts.

**Table 6:** Hemolytic and anti-hemolytic antioxidant activity of plant methanol extracts.

Plant Extract	IC_50_ (µg/mL)		
	DPPH	Hemolysis	AAPH
*J. spicigera*	15.68 ± 1.79 ^c^	642.10 ± 3.30 ^d^	16.22 ± 1.18 ^d^
*A. adstringens*	17.22 ± 2.02 ^c^	268.40 ± 13.90 ^a^	1.07 ± 0.31 ^a^
*S. mexicanum*	581.50 ± 1.09 ^j^	>1000 ^†^	8.26 ± 1.42 ^bc^
*A. ludoviciana*	89.90 ± 3.92 ^f^	>1000 ^†^	10.06 ± 0.76 ^c^
*H. inuloides*	61.62 ± 7.95 ^e^	427.20 ± 2.81 ^b^	1.09 ± 0.08 ^a^
*P. decompositum*	106.10 ± 1.06 ^g^	>1000 ^†^	1.18 ± 0.24 ^a^
*P. obtusifolium*	528.67 ± 25.78 ^i^	>1000 ^†^	28.63 ± 2.31 ^f^
*T. lucida*	550.85 ± 16.09 ^ij^	843.84 ± 31.93 ^f^	12.50 ± 1.14 ^c^
*J. dioica*	116.80 ± 1.70 ^h^	565.80 ± 2.07 ^c^	14.58 ± 0.48 ^d^
*M. tenuiflora*	2.86 ± 1.26 ^a^	>1000 ^†^	14.25 ± 1.58 ^d^
*A. mexicana*	565.98 ± 17.60 ^ij^	973.88 ± 38.46 ^g^	20.32 ± 0.21 ^e^
*C. citratus*	690.40 ± 26.37 ^k^	606.82 ± 19.12 ^cd^	6.40 ± 1.49 ^b^
*R. chalepensis*	859.85 ± 25.08 ^l^	738.73 ± 20.74 ^e^	5.66 ± 0.82 ^b^
*S. aspera*	29.21 ± 10.43 ^d^	262.80 ± 2.07 ^a^	0.88 ± 0.17 ^a^
*L. tridentata*	335.20 ± 1.60 ^i^	>1000 ^†^	155.57 ± 8.91 ^g^
C+	7.23 ± 0.03 ^b^	ND	316.14 ± 30.19 ^h^

Data are mean ± SD of the IC_50_ (µg/mL) for each evaluated extract. Different letters within the same column are significantly different, analyzed by the post hoc Tukey test (*p* < 0.05). C+: Positive control. ND: Not determined. ^†^ As IC_50_ was above 1000 µg/mL, these values were not considered for Tukey analysis.

## Data Availability

The datasets generated or analyzed during the present study are available from the corresponding author.

## Supplementary Materials

The following supporting information can be downloaded at: https://www.mdpi.com/article/10.3390/life13040958/s1, Phytochemical Screening Tests.
