# Supplementary material for: In Vitro Biological Activity and Lymphoma Cell Growth Inhibition by Selected Mexican Medicinal Plants
Source: Life (Basel). 2023 Apr 6;13(4):958. doi: 10.3390/life13040958 (PMC10143981; doi:10.3390/life13040958)
Supplement: Supplementary file 1 [file life-13-00958-s001.zip › life-2254797-supplementary.pdf]

Supplementary Material

Phytochemical Screening Tests

S1. Alkaloids (Dragendorff Test)

An amount of 1 mg of the extract was dissolved in 2 mL of methanol, and four drops of Dragendorff reagent were added; the test was considered positive if a persistent red-orange coloration was present. To prepare the reagent, two solutions were used: Solution A, which contained 0.85 g of $Bi(NO_3)_3$, which was mixed with 10 mL of CH_3COOH and 40 mL of water, and Solution B, which contained 8 g of KI dissolved in 20 mL of water. The reagent was prepared by mixing 5 mL of A, 4 mL of B, and 100 mL of water.

S2. Carbohydrates (Molish Test)

Molish's reagent (1% alpha-naphthol in ethanol) was added dropwise to 1 mg of the extracts, and then 2 mL of H_2SO_4 was added through the walls of the test tube. The test was considered positive when a purple-colored ring formed at the interface.

S3. Coumarin Test

An amount of 2 mg of the extracts was dissolved in 2 mL of methanol, and 10% NaOH was added dropwise. The test was considered positive if a yellow coloration was present and if it disappeared when the solution was acidified.

S4. Instaurations ($KMnO_4$ Test)

1 mg of the extracts was dissolved in 2 mL of methanol, and four drops of 2% $KMnO_4$ in water were added. The test was considered positive when discoloration or formation of a brown precipitate was observed, a result of the formation of MnO_2.

S5. Flavonoids (H_2SO_4 Test)

An amount of 1 mg of the extract was dissolved in 2 mL of H_2SO_4, and a positive result was indicated by yellow coloration for flavonoids, orange-cherry for flavones, red bluish for chalcones, and red-purple for quinones.

S6. Quinones (NaOH Test)

5 - 10 mg of the sample, 0.2 mL of ethanol, and 0.4 mL of a 5 % aqueous sodium hydroxide solution are introduced into a test tube. It is observed if there is color formation, and its ultraviolet spectrum is registered.

S7. Saponins ($NaHCO_3$ Test)

The aqueous solution of 10% $NaHCO_3$ was prepared, and then 2 mg of the extracts were dissolved in 2 mL of methanol, and four drops of concentrated H_2SO_4 were added. It was stirred slightly, and four drops of the $NaHCO_3$ solution were added. The appearance of bubbles and their permanence for more than 1 min indicated the presence of saponins.

S8. Sesquiterpene-Lactones (Baljet Test)

An amount of 2 mg of the extracts was dissolved in 2 mL of ethanol, and three drops of the mixed solution were added, with a positive result being indicated if it turned from orange to dark red. The 1:1 mixed solution consisted of Solution A, which contained 1% $C_6H_3N_3O_7$ in ethanol, and Solution B, which contained 10% NaOH.

S9. Sterols and Terpenes (Salkowski Test)

An amount of 1 mg of each extract was dissolved in 2 mL of chloroform, and subsequently, 2 mL of H_2SO_4 was added. A positive reaction was considered for sterols and methyl sterols when a red-brown ring was formed at the interface.

S10. Phenolic Compounds (Tannins) ($FeCl_3$ Test)

1 mg of the extracts was dissolved in 2 mL of methanol, and then four drops of 2.5% $FeCl_3$ in water were added. The appearance of a red, blue-violet, or green precipitate was considered positive.
